# Protective Effect of Arbidol Against Pulmonary Fibrosis and Sepsis in Mice

**DOI:** 10.3389/fphar.2020.607075

**Published:** 2021-01-27

**Authors:** Hailong Li, Rui Liu, Ruotong Zhang, Shanshan Zhang, Yiying Wei, Liang Zhang, Honggang Zhou, Cheng Yang

**Affiliations:** ^1^ The State Key Laboratory of Medicinal Chemical Biology, College of Pharmacy and Key Laboratory of Molecular Drug Research, Nankai University, Tianjin, China; ^2^ High-Throughput Molecular Drug Screening Centre, Tianjin International Joint Academy of Biomedicine, Tianjin, China; ^3^ Department of Thoracic Surgery, Tian Jin First Central Hospital, Tianjin, China

**Keywords:** arbidol, COVID-19, pulmonary fibrosis, cytokine storm, sepsis

## Abstract

From the perspective of epidemiology, viral immunology and current clinical research, pulmonary fibrosis may become one of the complications of patients with Coronavirus Disease 2019 (COVID-19). Cytokine storm is a major cause of new coronavirus death. The purpose of this study was to explore the effects of antiviral drug arbidol on cytokine storm and pulmonary fibrosis. Here, we use a mouse model of bleomycin-induced pulmonary fibrosis and a mouse model of fecal dilution-induced sepsis to evaluate the effects of arbidol on pulmonary fibrosis and cytokine storm. The results showed that arbidol significantly reduced the area of pulmonary fibrosis and improved lung function (reduced inspiratory resistance, lung dynamic compliance and forced vital capacity increased). Treatment with arbidol promoted reduced sepsis severity 48 h after sepsis induction, based on weight, murine sepsis score and survival rate. Arbidol observably alleviates inflammatory infiltrates and injury in the lungs and liver. Finally, we also found that arbidol reduced serum levels of pro-inflammatory factors such as TNF-α and IL-6 induced by fecal dilution. In conclusion, our results indicate that arbidol can alleviate the severity of pulmonary fibrosis and sepsis, and provide some reference for the treatment of cytokine storm and sequelae of pulmonary fibrosis in patients with COVID-19.

## Introduction

On December 30, 2019, 27 pneumonia cases linked to a seafood wholesale market were found in Wuhan, China, and promptly reported to the National Health Commission of China (Lake, 2020). A new type of coronavirus (HCoV), severe acute respiratory syndrome coronavirus-2 (SARS-CoV-2), was discovered through deep sequencing and analysis of lower respiratory tract samples (Gorbalenya et al., 2020; Zhu et al., 2020a). SARS-COV-2 is similar to SARS-COV and has sequence homology, but it spreads faster (Gorbalenya et al., 2020). As of November 30, SARS-COV-2 is still raging around the world, with a total of 63 million confirmed cases worldwide (Data comes from the World Health Organization, official notifications and authoritative media reports), but there are still no effective treatment drugs and methods.

Many critically ill patients and deceased patients have no serious clinical manifestations in the early stage of the virus infection, and only show mild fever, cough and other physical discomfort, but their bodily condition suddenly deteriorated during the treatment and turned into severe patients. Numerous severe COVID-19 patients will develop acute respiratory distress syndrome (ARDS) and multiple organ failure, which will eventually lead to death (Azkur et al., 2020; Yang et al., 2020). Dina Ragab and Harapan et al. (Harapan et al., 2020; Ragab et al., 2020) suggest that mortality in COVID-19 patients is related to the “cytokine storm” caused by the virus. Excessive production of proinflammatory cytokines lead to ARDS aggravation and extensive tissue damage, leading to multiple organ failure and death. In the case of COVID-19, clinical studies have concluded that the rapid deterioration of COVID-19 patients is closely related to the outbreak of cytokine storms in the body. Cytokine storm is an important cause of death. In addition, cytokine storms are a major cause of death in patients with SARS, MERS, and influenza (Liu et al., 2016; Channappanavar and Perlman, 2017; Rat et al., 2020). Therefore, inhibiting cytokine storms can effectively prevent death in COVID-19 patients. Sepsis refers to systemic inflammatory response syndrome (SIRS) caused by infection (Butler, 2015; Schlapbach et al., 2015). Sepsis, with its rapid development and high fatality rate, has always been one of the major difficulties in the study of acute illness (Li et al., 2015). The severity of sepsis infection is caused by a cascade reaction, which makes the automatic amplification of cytokines, and finally leads to a cytokine storm (Chousterman et al., 2017). Mouse models of sepsis are usually study cytokine storms. Song et al. (Qiu et al., 2019) found that regulatory T cells lacking Gpr174 reduced cytokine storms in septic mice. Tang et al. (Yao et al., 2017) revealed that α-lactose can reduce the cytokine storm by preventing TIM-3 signal transduction, thereby improving the survival rate of septic mice. Therefore, exploring the cytokine storm in sepsis mice has partial reference significance for the clinical cytokine storm.

Although the spread of the epidemic is still not ended, the sequelae of the epidemic are gradually emerging, and we need to explore in depth. At present, the familiar coronaviruses include SARS coronavirus, MERS and Novel Coronavirus. The study found that idiopathic pulmonary fibrosis (PF) was the most important cause of high mortality and low quality of life in patients infected with SARS coronavirus (Venkataraman and Frieman, 2017). Recently, through retrospective analysis of lung computed tomography imaging of 50 patients with COVID-19 pneumonia. It was found that the formation of fibrous streaks during rehabilitation (Xu et al., 2020). A meta-analysis found that viral infections increase the risk of idiopathic pulmonary fibrosis, and COVID-19 patients in the recovery period may cause additional complications due to PF (Sheng et al., 2020; Taz et al., 2020). Therefore, pulmonary fibrosis is likely to be one of the major complications in COVID-19 patients. The U.S. Food and Drug Administration (FDA) approved nintedanib and pirfenidone to inhibit the progression of PF (Raghu et al., 2015). They can only alleviate the decline in lung function and cannot reverse the fibrosis that has formed (Holtze et al., 2020). Therefore, it is very necessary to develop novel therapies for the treatment of PF and pay attention to the sequelae of SARS-COV-2 pulmonary fibrosis.

Arbidol (ethyl-6-bromo-4-[(dimethylamino) methyl]-5-hydroxy-1-methyl-2-[(phenylthio) methyl]-indole-3 carboxylate hydrochloride monohydrate, also known as UMifenovir), a broad-spectrum antiviral compound synthesized by the Russian Institute of Chemistry and Medicine in 1988, has been approved for the prevention and treatment of influenza A and B infections and post-influenza complications in China and Russia (Haviernik et al., 2018). Previous studies have shown that arbidol has an inhibitory effect on many global viruses with significant morbidity and mortality (Pécheur et al., 2016). Arbidol, oseltamivir, ribavirin and lopinavir (individualized or combined medication) are widely used in Chinese patients with COVID-19, but none of them have obvious effects. However, a retrospective study in a clinical trial found that in 220 non-emergency COVID 19 patients in Wuhan Dongxihu Hospital, China, arbidol could accelerate the fever recovery and virus clearance of respiratory specimens, especially in men, and also help shorten the length of hospital stay, And no obvious adverse reactions (Gao et al., 2020). However, the effect of arbidol on the cytokine storm and PF produced in patients with COVID-19 has not been reported.

Since our laboratory is not qualified for virus testing, in this study we used a fecal diluent-induced sepsis mouse model and a bleomycin-induced IPF mouse model to evaluate the effect of arbidol on cytokine storm and IPF, in order to provide reference for the clinical treatment and sequelae prevention of COVID-19.

## Materials and Methods

### Materials

Nintedanib (>99%) was purchased from HWRK Chem Co., Ltd. (Beijing, China). BLM was purchased from HANHUI Pharmaceuticals Co., Ltd (Shanghai, China). Cefpirome sulfate was obtained from Yuanye Bio-Technology Co., Ltd. (Shanghai, China). Arbidol (>98%) was purchased from Bidepharm (Shanghai, China).

### Experimental Animals

Male C57BL/6 mice of about 6–8 weeks and weighed (18 ± 4) g were provided by Beijing Academy of Military Sciences (Beijing, China). Mice had free access to food and water and housed in a room at a temperature of 20°–26°C and a humidity between 50 and 55%, with 12 h light/dark cycle. All experimental protocols were approved by the Animal Experiment Committee of Tianjin International Joint Academy of Biomedicine (approval no. SYXK (JIN) 2017-0003).

### Modeling and Animal Grouping

In bleomycin (BLM)-induced pulmonary fibrosis model, the mice were randomly divided into five groups (n = 8): Saline group, BLM only group (2 mg·kg^−1^), BLM with nintedanib group (100 mg·kg^−1^) and BLM with arbidol (100 and 200 mg·kg^−1^) treatment groups. At day 0, C57BL/6 mice were anesthetized by intraperitoneal injection of 10% chloral hydrate (5·ml/kg), and then intratracheal injection of 2 mg/kg BLM (Nippon Kayaku Co., Ltd. Tokyo, Japan). Saline group received normal saline by the same procedure. Normal saline, nintedanib and arbidol were applied intragastrically from day 7 to day 13.

In fecal dilution-induced sepsis model, the mice were randomly divided into five groups (n = 8): Saline group, 10% fecal dilution only group, fecal dilution with cefpirome sulfate (CS) group (100 mg·kg^−1^) and fecal dilution with arbidol (100 and 200 mg·kg^−1^) treatment groups. At day 0, Intraperitoneal injection of 10% fecal dilution was given to model group and treatment group to establish the sepsis models. For the treatment group, intraperitoneal injection of cefpirome sulfate (100 mg·kg^−1^) and arbidol (100 and 200 mg·kg^−1^) were given 2 h before fecal dilution injection. Saline group received normal saline by the same procedure.

### Histological

The heart, liver, spleen, lung and kidney tissues were fixed in 4% paraformaldehyde, embedded in paraffin and sectioned into 5 μm slices. Put the prepared slices in a 60°C oven for drying, and then dewax them as usual. Hematoxylin staining for 5–10 min, hydrochloric ethanol separation for 10–20 s. Then rinse with ultra-pure water for 15–20 min and dye with eosin for 3 min (H&E; Solarbio). After dehydration and transparency, add neutral gum to seal the slice. The histologic severity was quantified by Ashcroft scoring system (Ashcroft et al., 1988).

### Hydroxyproline Determination

The improved method of Dong et al. (Dong et al., 2015). was used to isolate the right lung tissue of mice with bleomycin pulmonary fibrosis and determine the level of Hydroxyproline.

### Pulmonary Function Test

The mice were kept supine and fixed on the operating table after anesthesia. When the trachea is exposed, insert the tube into the trachea and secure it with cotton thread. The pulmonary dynamic compliance (Cldyn) and forced vital capacity (FVC), expiratory resistance (Re) and inspiratory resistance (Ri) mice was measured after the mice were transferred to the body description platform.

### ELISA for the Detection of Inflammatory Factors

The levels of IL-6 and TNF-α in serum of fecal dilution-induced sepsis mice were determined by enzyme-linked immunosorbent assay (ELISA) kit. The experimental process strictly followed the instructions of the ELISA kit (Proteintech, United States).

### Statistical Analysis

All the data were analyzed using Graph Pad Prism 8.1 (Graph Pad Software, Inc., San Diego, CA) and expressed as mean ± SEM. All the data were statistically evaluated using one-way ANOVA for post-mortem multiple group comparisons. *p* < 0.05 was considered to be statistically significant.

## Results

### Arbidol Attenuates BLM-Induced Pulmonary Fibrosis in Mice

**FIGURE 1:**
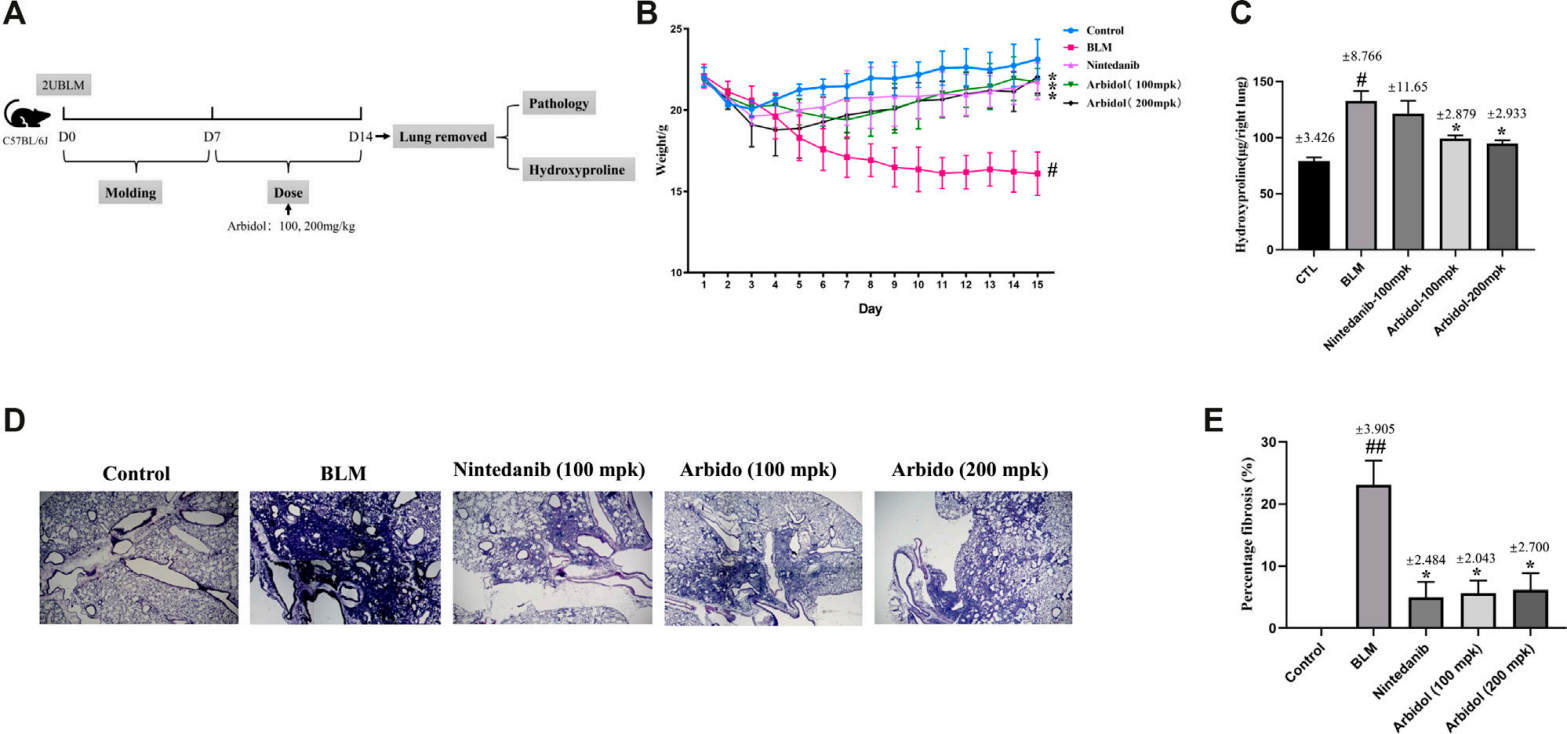
Effect of arbidol on BLM-induced pulmonary fibrosis in mice. **(A)** Experimental protocol of BLM-induced IPF in mice. **(B)** Arbidol significantly inhibited BLM-induced weight loss in mice. **(C)** Arbidol significantly inhibited the levels of hydroxyproline in the lung tissue of mice **(D)** HE staining was used to evaluate the effect of arbidol on BLM-induced pathological changes (20× magnification). **(E)** Percentage of fibrosis was quantified by Ashcroft score system. Values are presented as the mean ± SEM (n = 5), ^#^ *p* < 0.05, significantly different from control group; *p < 0.05 significantly different from model groups.

We first evaluated the effects of arbidol treatment on BLM-induced pulmonary fibrosis, and the experimental program is shown in Figure 1A. The change of body weight was displayed in Figure 1B. The body weight was decreased apparently with the treatment of BLM. As showed in Figure 1C, compared with saline group, the content of hydroxyproline in the right lung of mice in bleomycin group was significantly increased. In BLM with arbidol treatment groups, the hydroxyproline content decreased, indicating that arbidol inhibited ECM deposition. Histological changes of the lung were shown in Figures 1D,E, in the saline group, the lung tissue structure was normal without obvious inflammatory cell infiltration. After injection of bleomycin, the lung tissues of mice showed significant alveolar inflammation, and some alveolar changes and fibrosis occurred. After arbidol treatment, the tissue lesions were alleviated and the percentage fibrosis was observably reduced.

### Arbidol Alleviated BLM-Induced Pulmonary Function Impairment in Mice

**FIGURE 2:**
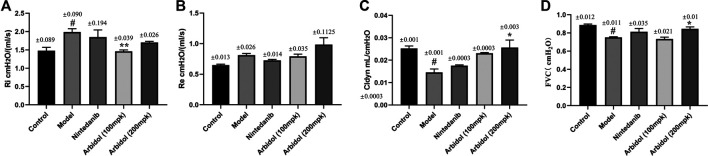
Effect of arbidol on BLM-induced pulmonary function impairment in mice. Lung function **(A)** (inspiratory resistance, RI) **(B)** (expiratory resistance, Re) **(C)** (dynamic lung compliance, Cldyn) and **(D)** (forced vital capacity, FVC) of the mice in the different groups described above were measured by pulmonary function apparatus. Values are presented as the mean ± SEM (n = 5), ^#^ *p* < 0.05, significantly different from control group; *p < 0.05, **p < 0.01 significantly different from model groups.

Pulmonary function determination is an important clinical examination of lung diseases and respiratory physiology. It is helpful for early detection of lung and airway lesions, assessing the severity and prognosis of the disease, and can also be used to evaluate the efficacy of drugs or other treatment methods. We have evaluated the effect of arbidol on BLM-induced pulmonary function impairment in mice. As showed in Figures 2A,B, compared with model group, arbidol significantly decreased Ri, but have no effect of Re. The results suggested that arbidol could reduce the airway resistance induced by BLM in mice, and thus alleviate the pulmonary function impairment caused by pulmonary fibrosis in mice. Cldyn is often used to detect the elastic resilience of lung tissue and determine the severity of obstructive lung disease. The decline in FVC is an alternative endpoint for death in IPF studies, and new drug development needs to demonstrate its efficacy against FVC under current treatment standards. As showed in Figures 2C,D, compared with the model group, arbidol significantly increased Cldyn and FVC. In conclusion, these data suggested arbidol could restore some lung function impairment indicators in pulmonary fibrosis mice.

### Arbidol Attenuates Fecal Dilution -Induced Sepsis in Mice

**FIGURE 3:**
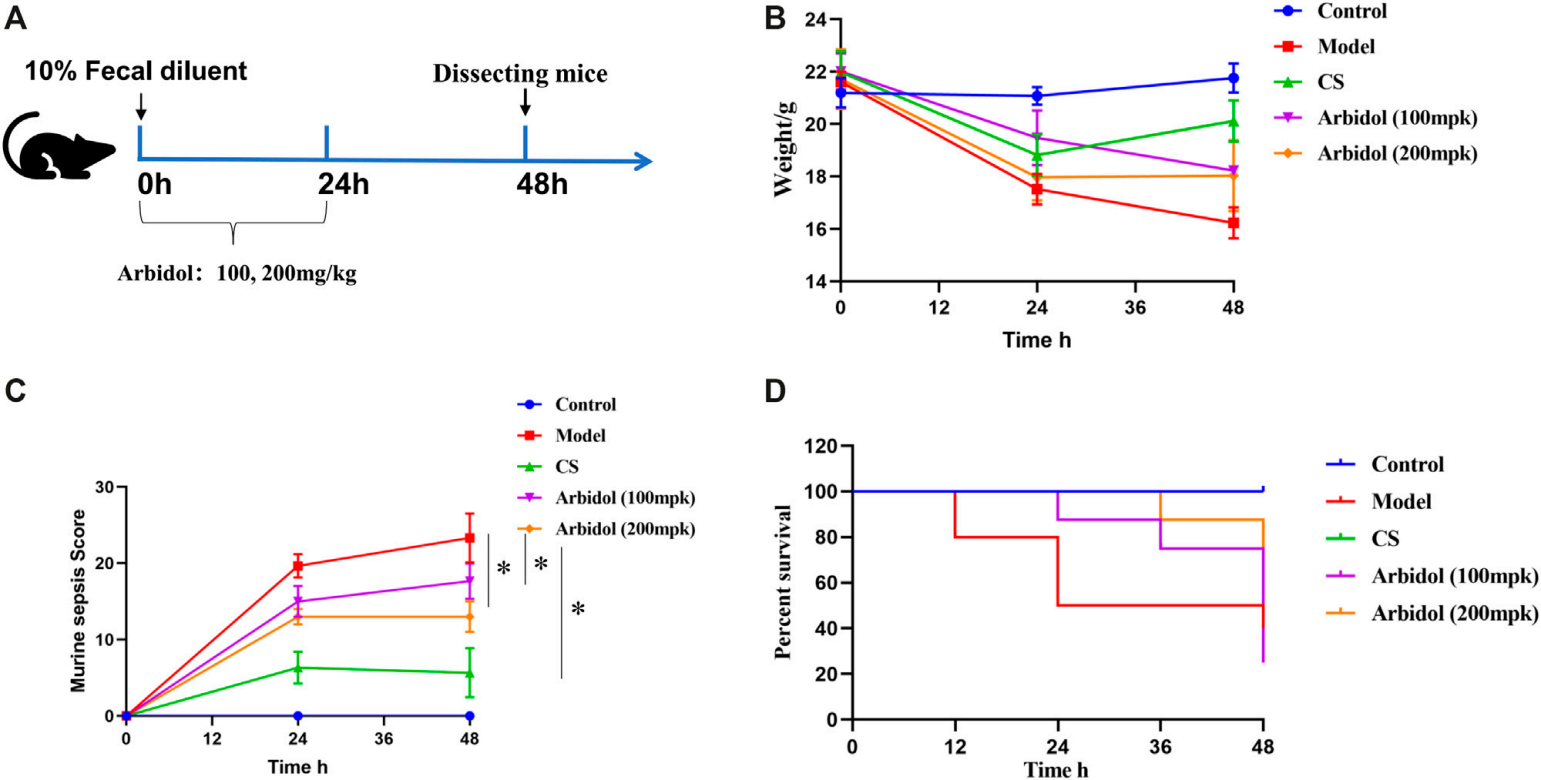
Effect of arbidol on fecal dilution -induced sepsis in mice **(A)** Experimental scheme of mice sepsis induced by fecal diluent **(B)** Changes in body weight in different groups of mice (Control, Model, cefpirome sulfate or CS, 100 and 200 mpk arbidol) **(C)** The effect of arbidol on murine sepsis score (MSS) of the different groups of mice described above **(D)** The effect of arbidol on the survival time of the different groups of mice described above. Values are presented as the mean ± SEM (n = 5), ^#^ *p* < 0.05, significantly different from control group; *p < 0.05, significantly different from model groups.

The experimental program is shown in Figure 3A. The change of body weight was displayed in Figure 3B. Sepsis-induced a decrease in body weights compared with the Control group, the body weight in mice treated with arbidol was higher than model group. The murine sepsis score (MSS) was tested according to the introduction of reference (Shrum et al., 2014). MSS values increased 24 and 48 h after induction of fecal diluent. However, the severity of sepsis evaluated by MSS was lower 24 and 48 h after sepsis induction in mice treated with arbidol (Figure 3C). A total of 40 mice were used in the survival experiments that were conducted independently over a period of 48 hour. As showed in Figure 3D, compared with the sepsis group, survival was improved in the arbidol treatment groups. The survival rate of the model group was 50% after 48 h induction of fecal diluent, whereas, during the same time period, the survival rates of arbidol group (100 and 200 mg·kg^−1^) were 80 and 90%, respectively.

### Arbidol Inhibits the Release of Pro-inflammatory Cytokines in Serum

**FIGURE 4:**
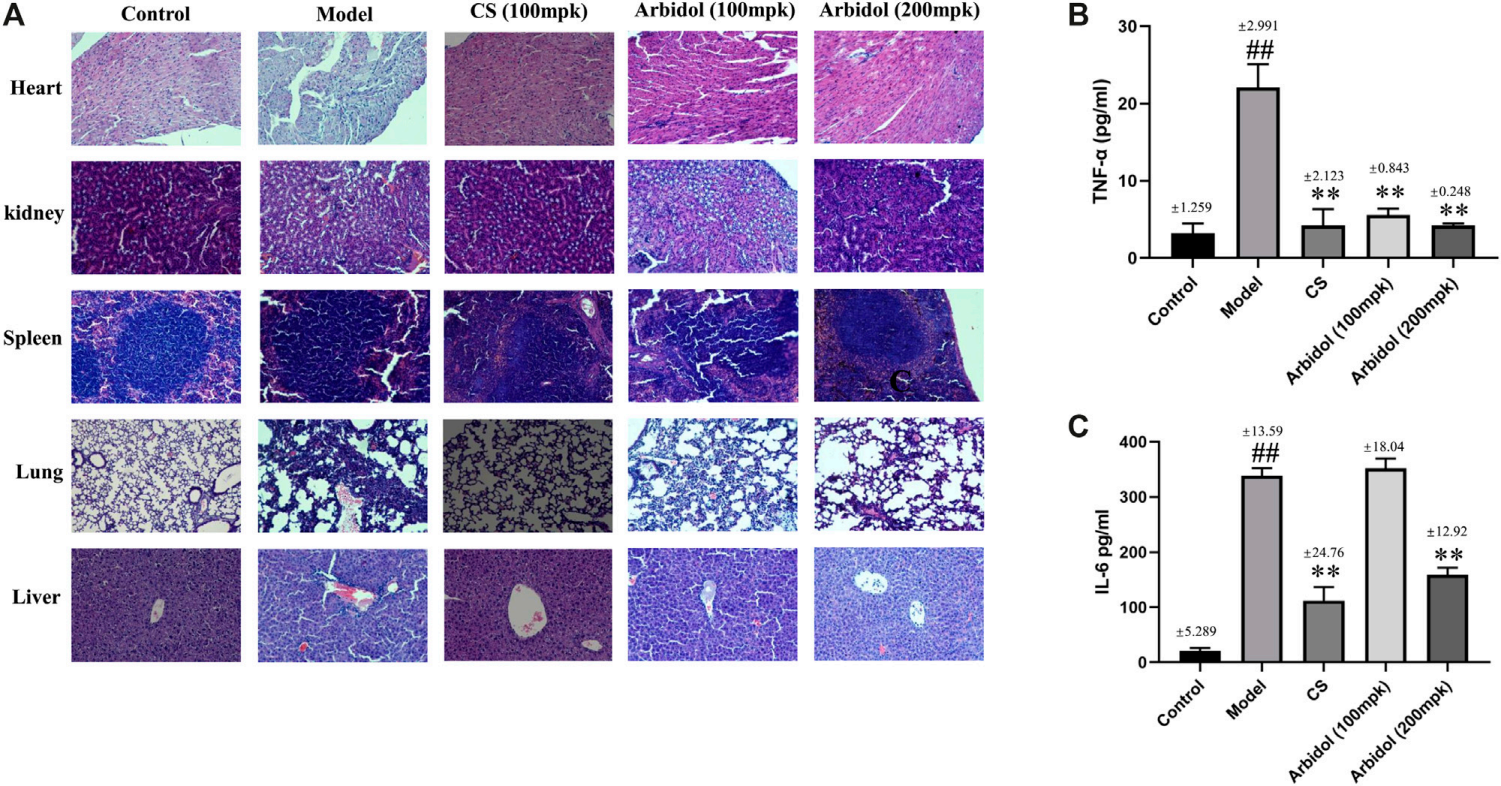
Effect of arbidol on serum proinflammatory cytokine expression. **(A)** HE staining was used to evaluate the effect of arbidol on fecal dilution -induced pathological changes (20× magnification). **(B,C)** Serum TNF-α and IL-6 were detected by ELISA. Arbidol significantly inhibits the levels of TNF-α and IL-6 in mouse serum. Values are presented as the mean ± SEM (n = 5), ^##^ *p* < 0.01, significantly different from control group; **p < 0.01, significantly different from model groups.

We also evaluated the effect of arbidol on sepsis induced tissue damage in mice. The histological changes are shown in Figure 4A. In the spleen of model group, the white pulp is significantly enlarged. In the lung of model group, mild alveolar space edema and leukocyte accumulation around pulmonary arterioles. In the liver and kidney of mice with fecal diluent, the tissue exhibited numerous inflammatory infiltrates. In the heart of model group, the cardiomyocytes are disorganized and loose. After arbidol intervention, the above lesions were alleviated.

As showed in Figures 4B,C, compared with the control group, content of inflammatory factors IL-6 and TNF-a in serum significantly increased after the injection of fecal diluent, while the content markedly decreased after the treatment of arbidol. These data suggested that arbidol could inhibit the elevation of serum inflammatory cytokines induced by sepsis.

## Discussion

Arbidol can inhibit the fusion of the viral lipid membrane and the cell body membrane under physiological conditions in the cell, and prevent the release of viral genetic material. At the same time, arbidol can enter the cell nucleus in its original form, change the permeability of the cell membrane, activate 2, 5-A synthetase, and induce the synthesis of 2, 5-oligoadenine nucleotides, which is an interferon-like effect, which leads to Disruption of cell metabolism, inhibit virus growth and cell proliferation (Boriskin et al., 2008). As a broad-spectrum antiviral, arbidol inhibits a variety of viruses, including parainfluenza, Lassa, Ebola, hepatitis B and C viruses (Blaising et al., 2014; Hulseberg et al., 2019). With regard to COVID-19, Xue et al. (Zhu et al., 2020b) showed that the duration of viral loss and hospital stay was significantly shorter in patients treated with arbidol, Lopinavir/Ritonavir, and recombinant interferon alpha-2B triple antiviral therapy compared to those treated without arbidol. Li et al. (Gao et al., 2020) analyzed the efficacy of arbidol and found that it accelerated fever recovery and virus clearance in respiratory tract samples. However, the effect of arbidol on sudden cytokine storms and pulmonary fibrosis in COVID-19 patients remains unknown. In this study, we used fecal diluent-induced septic mice and bleomycin-induced pulmonary fibrosis mice to partially simulate the symptoms of COVID-19 patients, including cytokine storm and sequelae of pulmonary fibrosis. The experimental results demonstrated that arbidol could significantly inhibit PF and sepsis in mice.

A single tracheal instillation of bleomycin in rodents is a widely used model for studying the occurrence of pulmonary fibrosis and evaluating the effects of anti-fibrosis treatments (Kolb et al., 2020). Bleomycin-induced pulmonary fibrosis model was in the early inflammatory stage from 0 to 7 days, and changed to fibrosis after 7 days (Plantier et al., 2018). This induction mode makes the timing of anti-pulmonary fibrosis treatment very important. Therefore, in order to accurately evaluate the anti-fibrosis effect of the drug, the intervention should be after 7 days without affecting the early inflammation. In this study, seven days after bleomycin-induced mice, arbidol treatment was started. Hydroxyproline (HYP) is mainly found in animal collagen and can enhance the elasticity of connective tissue. The content of hydroxyproline in the tissue reflects the catabolism of collagen. Therefore, accurate determination of the content of hydroxyproline in lung tissue can be directly and effectively determine the degree of development of pulmonary fibrosis (Srivastava et al., 2016). The results showed that arbidol could significantly reduce the area of fibrosis and the level of hydroxyproline in mouse lung tissue. Many types of lung function decline can affect the lower respiratory system, from the conducting trachea to the pulmonary vascular system. The clinical expression of pulmonary fibrosis is directly related to changes in lung function (Plantier et al., 2018). By testing the lung function of mice, it was found that arbidol could reduce the Ri of mice and increase Cldyn and FVC, but had no obvious effect on Re. Of course, we will continue to conduct in-depth research on the efficacy and mechanism of arbidol against pulmonary fibrosis.

Harapan et al. pointed out that SARS-CoV-2 infection induces an extremely active inflammatory response called cytokine storm, which subsequently leads to uncontrolled lung inflammation (Harapan et al., 2020). Another study also pointed out that COVID-19 infection is accompanied by an aggressive inflammatory response that releases a large amount of pro-inflammatory cytokines. This event is called a “cytokine storm” and is closely related to the mortality of patients with COVID-19 (Ragab et al., 2020). Sepsis, a complex disease, characterized by a host’s dysfunctional response to infection that leads to life-threatening multiorgan dysfunction (Dugar et al., 2020). Studies have found that the release of inflammatory cytokines, such as tumor necrosis factor (TNF-α) and interleukins (IL-1β and IL-6) increases, and promotes many immunopathological processes in sepsis, which are often referred to as “cytokines storm” (Ono et al., 2018; Hwang et al., 2020). As the most common source of infection in patients with sepsis comes from the abdominal cavity, in recent years, the abdominal infection model has been gradually used as a commonly used animal model of sepsis. Animal models of fecal peritonitis have been widely used in sepsis research (Dyson et al., 2011; Rudiger et al., 2013; Jeger et al., 2017; Sulzbacher et al., 2020). Since our laboratory cannot perform virus tests, in this study, we used fecal diluent-induced sepsis mice to simulate the cytokine storm symptoms of COVID-19 to a certain extent. The result revealed that arbidol notably attenuates fecal dilution -induced sepsis in mice. ELISA assay revealed that arbidol inhibits the release of pro-inflammatory cytokines (TNF-α and IL-6) in serum. Taken together, these data indicated that arbidol could inhibit the cytokine storm induced by fecal diluent in septic mice, and we hypothesized that arbidol may have a better inhibitory effect on cytokine storm in COVID-19 patients.

## Conclusion

In summary, our study found that arbidol can relieve PF and sepsis in mice. This study proves to a certain extent that arbidol may have a certain therapeutic effect in the cytokine storm that appears in the clinic of COVID-19 patients and the PF that appears during the rehabilitation period. Of course, the mechanism of action of arbidol on PF and sepsis needs to be further analyzed.

## Data Availability Statement

The original contributions presented in the study are included in the article/Supplementary Material, further inquiries can be directed to the corresponding authors.

## Ethics Statement

The animal study was reviewed and approved by Institutional Animal Care and Use Committee (IACUC) of Nankai University.

## Author Contributions

All authors listed have made a substantial, direct, and intellectual contribution to the work and approved it for publication.

## Funding

This work financial support by Tianjin Science and Technology Project (Grant no. 20ZXGBSY00050) and National Science & Technology Major Project “Key New Drug Creation and Manufacturing Program,” China (no. 2019ZX09201001-002.

## Conflict of Interest

The authors declare that the research was conducted in the absence of any commercial or financial relationships that could be construed as a potential conflict of interest.
